# Perceived Injustice as a Determinant of the Severity of Post-traumatic Stress Symptoms Following Occupational Injury

**DOI:** 10.1007/s10926-022-10056-5

**Published:** 2022-07-19

**Authors:** Antonina Pavilanis, Manon Truchon, Marie Achille, Pierre Coté, Michael JL Sullivan

**Affiliations:** 1 Department of Psychology, McGill University, 2001 McGill College, H3A 1G1 Montréal, QC Canada; 2 Université Laval, Montréal, QC Canada; 3 Université de Montréal, Montréal, QC Canada; 4 Ontario Tech University, Oshawa, ON Canada

**Keywords:** Perceived injustice, PTSD, PTSS, Pain, Disability, Injury

## Abstract

**Background:**

The present study assessed the role of perceived injustice in the experience and persistence of post-traumatic stress symptoms (PTSS) following work-related musculoskeletal injury.

**Methods:**

The study sample consisted of 187 individuals who were absent from work as a result of a musculoskeletal injury. Participants completed measures of pain severity, perceived injustice, catastrophic thinking, post-traumatic stress symptoms, and disability on three occasions at three-week intervals.

**Results:**

Consistent with previous research, correlational analyses revealed significant cross-sectional relations between pain and PTSS, and between perceived injustice and PTSS. Regression analysis on baseline data revealed that perceived injustice contributed significant variance to the prediction of PTSS, beyond the variance accounted for by pain severity and catastrophic thinking. Sequential analyses provided support for a bi-directional relation between perceived injustice and PTSS. Cross-lagged regression analyses showed that early changes in perceived injustice predicted later changes in PTSS and early changes in PTSS predicted later changes in perceived injustice.

**Conclusions:**

Possible linkages between perceived injustice and PTSS are discussed. The development of effective intervention techniques for targeting perceptions of injustice might be important for promoting recovery of PTSS consequent to musculoskeletal injury.

**Supplementary Information:**

The online version contains supplementary material available at 10.1007/s10926-022-10056-5.

## Introduction

Considerable research over the past two decades has addressed the relation between pain and post-traumatic stress symptoms (PTSS) [1]. The term PTSS has been used to refer to the presence of symptoms of post-traumatic stress disorder (PTSD) that do not meet all diagnostic criteria for PTSD [2]. PTSS can include a range of cognitive, emotional, behavioural and physiological symptoms such as intrusive thoughts, nightmares, flashbacks, emotional distress, avoidance behavior and hyperarousal [3, 4]. A significant relation between pain and PTSS has been reported in several populations including individuals with musculoskeletal pain [5], fibromyalgia [6], arthritis [7], cancer [8], burns [9] and chronic pain [10, 11].

Dominant conceptual frameworks of PTSD share in common the view that maladaptive cognitions play an important role in the experience of, and recovery from PTSD/PTSS [12, 13]. For example, Ehlers and Clark’s Cognitive Model of PTSD suggests that PTSS develop when trauma-related information is appraised in a manner that leads to a sense of serious and ongoing threat [12]. Numerous investigations have provided support for role of maladaptive cognitive processes in the development, severity and persistence of PTSS [14–17].

Recent research suggests that *perceptions of injustice* are a type of cognitive appraisal that might heighten the risk for the development of PTSS [18]. Adams [19] defined perceived injustice as “*a dissatisfied state of mind: a felt discrepancy between what is perceived to be and what is perceived should be”*. Perceived injustice has also been discussed as a psychological reaction to situations or events that are appraised as breaches of specific justice principles such as distributive or retributive justice [20, 21]. Several recent investigations have revealed significant relations between a number of injustice-related constructs (i.e., perceived injustice, moral injury, embitterment) and the severity of PTSS [18, 22–24].

The role of perceived injustice as a determinant of PTSS might be particularly relevant to individuals who have sustained a work-related musculoskeletal injury [25]. For many individuals, life following a work-related musculoskeletal injury will be characterized by significant and persistent physical and emotional suffering [26, 27]. Anecdotal accounts of injured workers reflect themes of perceived injustice associated with the claims handling process, the lack of choice of service providers and referrals for independent medical evaluations [28]. Franche et al. [29] have presented results showing that there are several aspects of compensation systems that can lead injured workers to experience the post-injury period as unjust. To date, the relation between perceived injustice and PTSS in individuals who have sustained disabling work injuries has not been examined.

Examining the role of perceived injustice as a determinant of PTSS in individuals who have sustained disabling work-injuries might have important clinical implications. Prolonged work absence following work injury remains a pressing social and economic problem [30]. As well, the number of claims for disability resulting from mental health problems such as PTSS continues to rise at an alarming rate [31, 32]. Theory and research suggest that the experience of persistent pain can contribute to the development of PTSS, even in the absence of a traumatic incident [5, 10, 33–35]. Given the high prevalence of pain symptoms following disabling work injury, injured workers might be particularly susceptible to developing PTSS. The experience of PTSS can add to the burden of disability associated with pain, in turn contributing to prolonged work absence [36, 37]. Research examining the processes implicated in the development or persistence of PTSS following work injury might help identify key targets for psychosocial interventions designed to improve recovery outcomes in individuals who sustain disabling work injuries.

The purpose of this prospective cohort study was to examine the role of perceived injustice as a predictor of the severity of PTSS in individuals who had sustained work-related musculoskeletal injuries. Individuals who recently sustained a disabling musculoskeletal injury were asked to complete measures of pain severity, perceived injustice, catastrophic thinking and PTSS on three occasions over a period of 6 weeks. Cross-sectional regression analyses were conducted to address the role of perceived injustice as a determinant of PTSS. Cross-lagged regression analyses were used to clarify the direction of influence between changes in perceptions of injustice and changes in PTSS. It was hypothesized that perceived injustice would contribute to the prediction of PTSS beyond the variance accounted for by injury characteristics and pain severity. It was also predicted that changes in perceived injustice would prospectively predict changes in the severity of PTSS.

## Method

### Participants

The participant sample comprised 187 individuals (92 men and 95 women) who sustained a disabling musculoskeletal injury in a work-related incident within 3 months of the date of enrolment in the study. Selection criteria included: musculoskeletal injury resulting from a sprain, strain or fall workplace incident, currently absent from work, and receiving or having applied for salary indemnity benefits. Participants were not considered for participation if they had sustained complex injuries also involving fracture, closed head injury, laceration, or organ damage.

## Measures

**Post-Traumatic Stress Symptoms.** The Post-Traumatic Stress Checklist (PCL) was used to assess post-traumatic stress symptoms. The PCL is a 17-item instrument where respondents indicate the degree to which they have been bothered by different symptoms of PTSD [38]. Ratings are made on a 5-point Likert scale with the endpoints (1) *not at all* and (5) *extremely*. Scores range from 17 to 85 where higher scores reflect more severe symptoms. A score of 44 has been recommended as the threshold for clinically significant post-traumatic stress symptoms [38]. Several studies have supported the reliability and validity of the PCL as a measure of PTSS [38, 39].

**Perceived Injustice.** The Injustice Experiences Questionnaire (IEQ) [25] was used to assess injury-related injustice appraisals. Participants were asked to rate the frequency with which they experienced each of 12 injustice-related cognitions on a 5-point scale, ranging from (0) *never* to (4) *all the time*. Previous research has supported the reliability and validity of the IEQ as a measure of injustice appraisals in individuals who have sustained musculoskeletal injuries [25, 40].

**Pain Severity.** A numerical rating scale (NRS) was used to assess of pain severity. Participants were asked to rate their current pain on an 11-point al NRS with the endpoints (0) no pain and (10) extreme pain. Previous research has supported the use of NRS pain scales as reliable and valid measures of pain severity [41].

**Number of Pain Sites.** Participants were asked to report the number of body areas where they experienced pain. Number of pain sites represents the total number of painful body areas reported by participants and was included as an index of injury severity. Previous research has shown that multi-site pain is a better predictor of work-disability than pain severity, and is associated with several markers of delayed recovery [42, 43].

**Pain Catastrophizing.** The Pain Catastrophizing Scale (PCS) was used to assess catastrophic thoughts associated with pain [44]. Participants were asked to rate the frequency with which they experienced each of 13 thoughts and feelings associated with their pain. Ratings were made on a 5-point scale, ranging from (0) *never* to (4) *all the time.* The PCS has been shown to have high internal consistency and to be associated with heightened pain and disability in patients with a range of health and mental health conditions [22, 24, 30, 31].

**Disability.** The Pain Disability Index (PDI) [45] was used as a self-report measure of disability. Participants rated their level of pain-related disability in 7 different areas of daily living (home/family, social, recreational, occupational, sexual, self-care, life support). Research has supported the reliability and validity of the PDI [46].

## Procedure

Participants were recruited through ads placed in 24 primary care physiotherapy clinics in the greater Montreal region. Participants were also recruited through ads placed on Facebook. Study advertisements solicited individuals who had sustained a disabling workplace musculoskeletal injury to the back or neck and were currently absent from work. Recruitment for this study began in March of 2019 and ended in July of 2021.

Prospective participants were interviewed by telephone to determine whether they met selection criteria for the study. Individuals who met selection criteria were provided with a web address and were guided through the setup of their study account. Participants signed a web-based consent form as a condition of enrolment. REDCap [47] was used as the questionnaire administration platform. Participants completed online questionnaires on three separate occasions at three-week intervals over a 6-week period. Participants received a compensation of 75$ for their involvement in the study.

This study received ethical approval from the Institutional Review Board of McGill University. The Strengthening the Reporting of Observational studies in Epidemiology (STROBE) guidelines for observational studies was used as a reference for reporting the study [48].

## Data Analytic Approach

Preliminary analyses examined whether scores on dependent measures varied a function of recruitment method (i.e., physical therapy clinics vs. Facebook). No significant differences were found on any of the study variables. As such, the results are presented collapsed across recruitment method. A table comparing the scores on dependent measures between participants recruited in physical therapy clinics and Facebook is provided as supplementary material.

Descriptive statistics were computed on all study variables. Tests of mean differences were used to compare women and men on continuous variables. Chi-square analyses were used to compare women and men on categorical variables. Correlational analyses were used to examine relations among measures of perceived injustice, pain severity, number of pain sites, PTSS, catastrophic thinking, and disability.

A cross-sectional hierarchical regression analysis was used to examine the value of perceived injustice as a predictor of PTSS at the time of enrolment. In light of previous research showing significant associations between level of perceived injustice, pain severity and pain catastrophizing, the NRS, number of pain sites and the PCS were included as covariates in the regression analysis [25]. Power analysis was conducted with Power and Precision software [49]. Assuming a medium effect size for the relation between perceived injustice and PTSS, in a single sample model with 6 covariates (age, sex, time since injury, number of pain sites, pain severity, pain catastrophizing), with N = 187 and alpha set at 0.01, power exceeds 0.85. The assumption of medium effect sizes is supported by the research that has been conducted to date [25, 50].

Cross-lagged regression analyses were used to examine the sequential relations between early and late changes in levels of perceived injustice and the severity of PTSS. Cross-lagged regression analysis involves comparing the correlation between Variable A at Time 1 (i.e., perceived injustice) and Variable B (i.e., PTSS severity) at Time 2 with the correlation between Variable B (i.e., PTSS severity) at Time 1 and Variable A (i.e., perceived injustice) at Time 2 [51]. This analytic approach addresses whether a change in one variable, that precedes a given change in a second variable, predicts the subsequent change in the second variable. Tolerance coefficients for all variables included in the regression analyses were greater than 0.60 indicating no multicollinearity. Analyses were conducted with SPSS Version 27.

## Results

### Sample Characteristics

Means and standard deviations on all study variables are presented stratified by sex in Table 1. The mean age of the sample was 36.71 years (range = 18 to 61 years), and the mean duration of absence from work at the time of enrolment was 7.20 weeks (range = 4 to 12 weeks). Slightly more than half of the participants were married or living common law (53%), and the majority had completed high school (82%). The majority of participants were using some form of medication for pain control.

**Table 1:** Sample Characteristics at the Time of Enrolment

Variable	Men		Women		
	**N = 92**		** N = 95**		
	**Means**	**SD**	**Means**	**SD**	**P-value**
**Age, years (SD)**	37.1	10.4	36.6	9.8	ns
	**n**	**%**	**n**	**%**	
**Marital Status**					ns
** Single**	36	39%	24	25%	
** Married or common law**	45	49%	54	57%	
** Separated or divorced**	10	11%	17	18%	
** Widowed**	1	1%	0	0%	
**Education**					0.01
** Less than High School**	22	24%	11	12%	
** High School/Trade School**	50	54%	43	45%	
** College**	11	12%	29	30%	
** University**	9	10%	12	13%	
**Occupation**					0.02
** Labour/Trade**	48	52%	31	33%	
** Health/Nursing**	23	25%	37	39%	
** Admin/clerical**	14	15%	19	20%	
** Sales**	4	5%	8	8%	
** Education**	3	3%	0	0%	
**Pain sites**					
** Back**	86	93%	90	95%	ns
** Neck**	59	64%	76	80%	0.05
** Upper limb**	41	45%	62	65%	0.05
** Lower limb**	11	12%	10	11%	ns
**Medication**					
** NSAIDs**	48	53%	47	50%	ns
** Anti-inflammatory**	64	70%	74	87%	ns
** Opioid**	17	18%	20	21%	ns
** Psychotropic Med**	14	15%	33	35%	0.05
**Current treatment**					
** Physiotherapy**	83	90%	79	83%	ns
** Occupational Therapy**	11	12%	12	13%	ns
** Psychology**	6	7%	3	3%	ns
**Weeks Since Injury**					ns
** 0–4 weeks**	26	28%	17	18%	
** 5–8 weeks**	45	49%	45	47%	
** 9–12 weeks**	21	23%	33	35%	
	**Means**	**SD**	**Means**	**SD**	
**Clinical Measures**					
**NRS**_**pain**_ **(0–10)**	4.74	1.9	5.32	1.5	0.05
**PCL**_**ptss**_ **(17–85)**	25.2	20.9	35.5	21.5	0.01
**IEQ**_**injustice**_ **(0–48)**	19.6	10.3	22.1	9.2	0.07
**PCS**_**catastrophizing**_ **(0–52)**	19.9	11.1	21.9	10.1	0.21
**PDI**_**disability**_ **(0 – 70)**	25.7	11.4	26.4	9.1	0.64

Note: *N* = 187. SD = Standard Deviation. The sum of percentages, within sex, for pain sites, medication and concurrent treatment exceeds 100% since participants could be experiencing pain in more than one site, and could have been prescribed more than one treatment.

Women (M = 5.3, SD = 1.5) rated their pain as more severe than men (M = 4.7, SD = 1.9), t (185) = 2.3, p < .05. Women (M = 35.5, SD = 21.5) also reported more severe PTSS than men (M = 25.2, SD = 20.9), t (185) = 5.1, p < .001. Women were more likely than men to have completed a college degree. Women were over-represented in health/nursing occupations and men were over-represented in labour occupations. Women were more likely than men to report neck and upper limb pain.

Based on test scores at the time of enrolment, the study sample would be characterized as experiencing moderate to severe symptoms of pain [53] and PTSS of moderate severity [38]. At baseline, approximately one third of the sample (33%) scored above the recommended PCL cut score for clinically significant PTSS [38]. At the last test point, 15% of the sample scored above clinical threshold on the PCL.

## Correlates of Perceived Injustice at Time of Enrolment

Pearson correlations were computed to examine inter-relations among scores on measures of PTSS (PCL), pain severity (NRS), number of pain sites, perceived injustice (IEQ), pain catastrophizing (PCS) and disability (PDI), at the time of enrolment (Table 2). Significant correlates of the PCL included pain severity (r = .28, p < .01), number of pain sites (r = .26, p < .01), perceived injustice (r = .62, p < .01), pain catastrophizing (r = .59, p < .01), and disability (r = .33, p < .01). Consistent with previous research, perceived injustice and pain catastrophizing were also significantly correlated with pain severity.

**Table 2:** Correlates of Post-Traumatic Stress Symptoms

Variable	1	2	3	4	5
**1 PCL**					
**2 NRS**	0.28**				
**3 Pain sites**	0.26**	0.20**			
**4 IEQ**	0.62**	0.19**	0.29**		
**5 PCS**	0.59**	0.26**	0.17*	0.68**	
**6 PDI**	0.33**	0.29**	0.18*	0.18*	0.28**

Note: N = 187. PCL = Post-Traumatic Stress Checklist; NRS = Numerical Rating Scale; Pain Sites = Number of pain sites; IEQ = Injustice Experiences Questionnaire; PCS = Pain Catastrophizing Scale; PDI = Pain Disability Index. * = p < .05; ** = p < .01 level (2-tailed)

## Perceived Injustice as a Predictor of Post-traumatic Stress Symptom Severity: Cross-sectional Relations

A cross-sectional hierarchical regression analysis was conducted to evaluate whether perceived injustice contributed to the severity of PTSS beyond the variance accounted for by pain/injury-related variables and pain catastrophizing. As shown in Table 3, age, and sex were entered in Step 1 of the analysis but did not contribute significantly to the prediction of the severity of PTSS, F _change_ (2, 184) = 1.4, p = .24. Weeks since injury, pain severity and number of pain sites were entered in Step 2 of the analysis and made a significant contribution to the prediction of the severity of PTSS, F _change_ (3, 181) = 9.1, p < .001. The PCS was entered in Step 3 of the analysis and made a significant contribution to the prediction of the severity of PTSS, F _change_ (1, 180) = 80.1, p < .001. The IEQ was entered in the final step of the analysis and made a significant contribution to the prediction of the severity of PTSS, beyond the variance accounted for by variables already entered in the regression equation, F _change_ (1, 179) = 21.4, p = .002. Examination of the standardized beta weights from the final regression equation revealed that only pain severity (β = 0.12, p < .05), pain catastrophizing (β = 0.31, p < .01), and perceived injustice (β = 0.36, p < .01) contributed significant unique variance to the prediction of the severity of PTSS.

**Table 3:** Hierarchical Regression Predicting PTSD Symptom Severity

	β	u- β (95%CI)	R^2^_change_	F_change_ (df)	p
**Dependent = PCL**					
**Step 1**			.06	1.4 (2, 184)	.24
Age	− .02	− .01 (-.23 − .22)			
Sex	− .08	-4.00 (-8.9 − .91)			
**Step 2**			.13	9.1 (3, 181)	.001
Weeks since injury	− .02	.67 (-.15 − 1.5)			
NRS pain	.12*	1.47 (.06–2.8)			
Number pain sites	.09	1.77 (-1.4–3.7)			
**Step 4**			.26	80.1 (1, 180)	.001
PCS	.31**	.63 (.33 − .93)			
**Step 5**			.06	21.4 (1, 179)	.001
IEQ	.36**	.80 (.47–1.13)			

Note: N = 187. NRS = Numerical rating scale; PCS = Pain Catastrophizing Scale; IEQ = Injustice Experiences Questionnaire. β = Standardized beta weight: u- β = Unstandardized beta weight: Beta weights are from the final regression equation. * p < .05; ** p < .01

## Temporal Relations Between Perceived Injustice and the Severity of PTSS

A cross-lagged regression analysis was used to examine the temporal relations between changes in perceptions of injustice and changes in the severity of PTSS. Standardized residual change scores were computed for early (i.e., Time 1 – Time 2) and late (i.e., Time 2 – Time 3) changes in perceived injustice and PTSD symptom severity. A hierarchical regression analysis was used to examine the unique contributions of early change in perceived injustice to the prediction of late change in the severity of PTSS (Table 4). To control for the potential R inflation effects of synchronous and autocorrelations, early change in the severity of PTSS, and late change in perceived injustice were entered as covariates in Step 1 of the analysis. Early change in pain severity was also included as a covariate in Step 1 of the analysis. Early change in perceived injustice was entered in Step 2 of the analysis and contributed significant variance to the prediction of late change in the severity of PTSS, R^2^ change = 0.02, p < .02.

**Table 4:** Cross-Lagged Regression Analyses Examining Temporal Relations Between Changes in Perceived Injustice and PTSS Severity****alignment of decimals would be a cleaner look in the Tables****

			β		u- β (95%CI)		R^2^_change_		F_change_ (df)			p		
**Dependent = ΔPCL**_**2 − 3**_														
**Step 1**							0.13		9.7 (3,183)		0.001			
	ΔPCL_1 − 2_		-0.27**		− 0.28 (-0.42 - − 0.12)									
	ΔIEQ-_2−3_		0.29**		0.04 (0.02 − 0.06)									
	ΔNRS-_1−2_		0.04		0.04 (0.01 − 0.10)									
**Step 2**							0.02			5.7 (1,182)	0.02			
	ΔIEQ_1 − 2_		0.17*		0.17 (0.02 − 0.31)									
**Dependent = ΔIEQ**_**2 − 3**_														
**Step 1**							0.08			5.1 (3,183)	0.002			
	ΔIEQ_1 − 2_		-0.15*		− 0.16 (-0.31 − 0.01)									
	ΔPCL_2 − 3_		0.31**		0.31 (0.16 − 0.46)									
	ΔNRS-_1−2_		0.01		0.01 (-0.14 − 0.14)									
**Step 2**							0.03			4.4 (1,182)	0.03			
	ΔPCL_1 − 2_		0.16*		0.17 (0.01 − 0.32)									

Note: N = 187. PCL; Post-Traumatic Stress Checklist, IEQ; Injustice Experiences Questionnaire. β; standardized beta weight, u-β; unstandardized beta weight, df; degrees of freedom. Beta weights are from the final regression equation

*p < .05,** p < .01

A second hierarchical regression analysis was conducted to examine the unique contributions of early change in the severity of PTSS to the prediction of late change in perceived injustice. Early change in perceived injustice and late change in PTSS were entered in Step 1 of the analysis to control for the R inflation effects of synchronous and autocorrelations. Early change in pain severity was also included as a covariate in Step 1. Early change in the severity of PTSS was entered in Step 2 of the analysis contributed significant variance to the prediction of late change in the severity of PTSS, R^2^ change = 0.03, p < .03.

## Discussion

The present study addressed the relation between perceived injustice and PTSS in individuals who had sustained disabling musculoskeletal injuries. The findings of the present study are consistent with previous research showing that scores on a measure of pain severity were correlated with scores on a measure of PTSS [54]. The findings are also consistent with previous research showing that perceptions of injustice are correlated with PTSS [55]. The results of this study extend those of previous research in showing that PTSS are prevalent following work-related musculoskeletal injury and that perceptions of injustice contribute unique variance to the prediction of PTSS. Additionally, the study provided support for a bi-directional temporal relation between perceptions of injustice and PTSS.

In the present study, 15% of the sample reported clinically significant PTSS that persisted throughout the study period. Previous research has shown that PTSS adds to the burden of disability associated with musculoskeletal injury [56, 57]. Individuals with PTSS report decreased involvement in family, social, recreational, and occupational activities [31, 58, 59]. There are also indications that the presence of PTSS can contribute to delayed recovery of musculoskeletal injuries [60, 61]. Screening for PTSS is not part of routine practice in primary care and consequently, the majority of cases of PTSS are likely to be undetected and untreated [62]. The failure to detect clinically significant PTSS might be particularly likely in cases of musculoskeletal injury, where characteristics of the injury incident would not necessarily meet diagnostic criteria for a ‘traumatic event’. Researchers have called for greater attention to detection and treatment of PTSS in individuals seeking care for pain-related conditions [61, 63].

The findings of the present study join a growing body of research showing a significant relation between pain severity and PTSS in individuals with pain conditions [10, 54, 64]. Several explanations have been put forward to account for the relation between pain and PTSS. In the case of physical trauma (e.g., explosions, vehicle crash), it has been suggested that the injury incident could simultaneously give rise to symptoms of pain and PTSS [33, 65]. It has also been suggested that chronic pain and PTSS might share common vulnerability factors such as anxiety sensitivity or catastrophic thinking [35, 66], or might be mutually maintained [34].

The results of the present study are also consistent with previous research showing significant relations between perceptions of injustice and the severity of pain symptoms [18, 67–69]. To date, the relation between perceived injustice and adverse pain outcomes has been demonstrated in individuals suffering from a wide range of painful conditions including whiplash injury [18], low back pain [25], catastrophic injury [70], fibromyalgia [71], sickle cell disease [72], osteoarthritis [73], rheumatoid arthritis [74], traumatic brain injury [75], and multiple sclerosis [76]. It has been suggested that perceptions of injustice might trigger a cascade of cognitive (i.e., rumination), emotional (i.e., anger), behavioural (i.e., pain behaviour) and physical (i.e., muscle tension) reactions that impede recovery of pain conditions [77].

Only a few studies have examined the relation between perceptions of injustice and PTSS in individuals with pain conditions. Sullivan et al. [18] reported the results of a study examining the relation between pain severity and PTSS in a sample of individuals with whiplash symptoms enrolled in a multidisciplinary pain treatment program. Their findings showed that high scores on a measure of perceived injustice were associated with the persistence of PTSS through the course of the treatment program. Linnemorken et al. [10] reported that perceived injustice was positively correlated with a measure of PTSS in a sample of patients with long-standing chronic pain. To our knowledge, the present study is the first to report on the relation between perceived injustice and PTSS in a sample of workers with musculoskeletal injuries in the early stages of recovery.

In the present study, the results of a regression analysis revealed that perceived injustice contributed to the prediction of post-traumatic stress symptoms, independent of the variance accounted for by demographic and injury-related variables, pain severity and catastrophic thinking. Of interest was that perceived injustice remained a significant predictor of PTSS even when controlling for pain severity and pain catastrophizing. The latter finding suggests that perceived injustice impacts on PTSS through avenues that are distinct from those by which pain or catastrophizing might be contributing to PTSS.

Although the independent contribution of perceived injustice to PTSS in the cross-sectional regression was modest (6%), it is important to note that the analytic approach taken in this study was very conservative. The order of entry of variables in the regression analyses proceeded from the assumption that the severity of symptoms of pain and catastrophic thinking had theoretical primacy, and as such, were entered in an earlier step of the analysis. Given that characteristics of the injury context might have been sufficient to elicit injustice appraisals, there could also be grounds for assigning theoretical primacy to perceived injustice. If no assumptions are made about theoretical primacy, and the contributions of perceptions of injustice are assessed independently, univariate analyses reveal that perceived injustice accounted for approximately 38% of the variance in the severity of PTSS.

This is the first study to show a temporal or sequential relation between changes in changes in perceived injustice and changes in PTSS. The results of the cross-lagged regression analyses showed that early changes in perceived injustice predicted subsequent changes in post-traumatic stress symptoms, and early changes in post-traumatic stress symptoms predicted subsequent changes in perceived injustice. The findings are consistent with conceptual frameworks that propose that suffering and loss can play a causal role in giving rise to perceptions of injustice [78]. Symptoms of distress and disability associated with PTSS might be experienced as undeserved suffering, in turn eliciting injustice appraisals [78]. The findings are also consistent with conceptual models that propose a causal role of maladaptive cognitive processes in the development of PTSS [12]. It is possible that ruminative thinking associated with perceived injustice might accentuate emotional impact of the injury, increasing the probability of the onset or persistence of PTSS [79].

The bi-directional relation between perceived injustice and PTSS might help explain, at least in part, the transition to chronicity of many individuals who sustain workplace musculoskeletal injuries. Whether triggered by the severity or persistence of pain symptoms [78], or by factors associated with the claims process [80], post-injury perceptions of injustice could increase the probability of the development of PTSS. In turn, the experience of PTSS might reinforce the injured worker’s perceptions of injustice. Through a recursive process, perceptions of injustice and PTSS might become mutually maintaining. Since perceptions of injustice and PTSS have been linked to delayed recovery, the presence of both might be associated with particularly poor recovery outcomes. Women might be at higher risk for problematic recovery since they are likely to experience more severe pain and PTSS following injury. As with the relation between pain and PTSS, there is a basis for considering that the bi-directional relation between perceived injustice might be the result of shared vulnerability factors. Emotional factors such as anger or depression, that have been associated with both perceived injustice and PTSS are potential candidates as shared vulnerability factors [81, 82]. Future research will be needed to clarify the pathways linking perceived injustice and PTSS.

The findings of the present suggest that intervention approaches aimed either at preventing the emergence of perceptions of injustice, or reducing post-injury perceptions of injustice, might contribute to more positive recovery outcomes in individuals experiencing PTSS consequent to a work-related musculoskeletal injury. Indeed, emerging research suggests that treatment-related reductions in perceived injustice prospectively predict reductions in emotional distress and disability [83, 84]. Sullivan et al. [83] have described an intervention approach that focuses on the identification of sources of injustice, combined with problem-resolution strategies to reduce the number of factors that are contributing to the individual’s sense of injustice. Clinical researchers have also discussed the potential of intervention approaches such as Acceptance and Commitment Therapy and Adaptive Disclosure as treatments that could be beneficial in reducing the negative impact of perceived injustice on recovery outcomes [85, 86]. In light of the consistency with which perceptions of injustice have been linked to adverse recovery outcomes, a systematic effort to evaluating the effectiveness of different approaches to reducing perceptions of injustice appears warranted.

Some degree of caution must be exercised in the interpretation of the study findings. First, PTSS were assessed by self-report questionnaire as opposed to structured diagnostic interview. As such, it is unclear whether participants who scored within the clinical range on the PCL met diagnostic criteria for (PTSD). Confidence in the pattern of results reported here must await replication in a sample of individuals with musculoskeletal injuries for whom a diagnosis of PTSD has been confirmed. As well, all participants were working full time prior to the current period of work absence and were receiving salary indemnity from a work injury insurer. These sample characteristics necessarily have implications for the generalizability of findings. The modest unique relations between changes in perceived injustice and changes in PTSS also warrant reflection in evaluating the clinical implications of the findings. It is also important to consider that several condition-related, treatment-related and contextual factors that have been shown to influence recovery outcomes in individuals exposed to physical or emotional trauma were not assessed [65, 87, 88]. Whether the contribution of perceived injustice to PTSS is independent of the contributions of other PTSS-relevant factors remains to be clarified by future research.

In spite of these limitations, the results of the present study suggest that perceptions of injustice might play a role in the etiology of mental health problems consequent to work-related musculoskeletal injuries. The findings suggest that the perceptions of injustice are prevalent in the early stages of recovery following musculoskeletal injury and might be causally related to more severe and persistent PTSS. The development and evaluation of intervention techniques for targeting perceptions of injustice might be important for promoting recovery of PTSS, and in turn, foster more timely resolution of musculoskeletal injuries.

## Electronic supplementary material

Below is the link to the electronic supplementary material.

Supplementary Material 1
