# Supplementary material for: Perceived Injustice as a Determinant of the Severity of Post-traumatic Stress Symptoms Following Occupational Injury
Source: J Occup Rehabil. 2022 Jul 19;33(1):134–44. doi: 10.1007/s10926-022-10056-5 (PMC10025196; doi:10.1007/s10926-022-10056-5)
Supplement: Supplementary file 1 — Supplementary Material 1 [file 10926_2022_10056_MOESM1_ESM.docx]

**Supplementary Material Table 1**

**Sample Characteristics as a Function of Recruitment Approach**

| **Variable** | **Physiotherapy** | | **Facebook** | |  |
| --- | --- | --- | --- | --- | --- |
|  | **N = 112** | | **N = 75** | |  |
|  | **Means** | **SD** | **Means** | **SD** | **P-value** |
| **Age, years (SD)** | 36.8 | 10.1 | 36.6 | 10.2 | .87 |
| **Weeks Since Injury** | 7.1 | 2.9 | 7.3 | 2.9 | .76 |
| **NRS _pain_ (0 – 10)** | 5.1 | 1.7 | 5.0 | 1.8 | .89 |
| **PCL _ptss_ (17 – 85)** | 31.3 | 21.7 | 29.1 | 21.9 | .50 |
| **IEQ _injustice_ (0 – 48)** | 21.9 | 9.2 | 19.4 | 10.6 | .08 |
| **PCS _catastrophizing_ (0 – 52)** | 21.8 | 10.8 | 19.6 | 10.3 | .16 |
| **PDI _disability_ (0 – 70)** | 25.9 | 9.8 | 26.4 | 11.1 | .74 |

Note: *N*=187 . SD = Standard Deviation. NRS = Numerical Rating Scale; PCL = Post-Traumatic Stress Checklist; IEQ = Injustice Experiences Questionnaire; PCS = Pain Catastrophizing Scale; PDI = Pain Disability Index.
